# Mutations acquired by hepatocellular carcinoma recurrence give rise to an aggressive phenotype

**DOI:** 10.18632/oncotarget.14248

**Published:** 2016-12-27

**Authors:** Ji-Hye Choi, Min Jae Kim, Yong Keun Park, Jong-Yeop Im, So Mee Kwon, Hyung Chul Kim, Hyun Goo Woo, Hee-Jung Wang

**Affiliations:** ^1^ Department of Physiology, Ajou University School of Medicine, Suwon, Korea; ^2^ Department of Biomedical Science, Graduate School, Ajou University, Suwon, Korea; ^3^ Developmental Therapeutics Branch, Center for Cancer Research, National Cancer Institute, National Institutes of Health, Bethesda, Maryland, United States; ^4^ Department of Surgery, Catholic Kwandong University International St. Mary's Hospital, Incheon, Korea; ^5^ Department of Surgery, Ajou University School of Medicine, Suwon, Korea

**Keywords:** RNA-Seq, GOLGB1, SF3B3, recurrence, mutation

## Abstract

Recurrence of hepatocellular carcinoma (HCC) even after curative resection causes dismal outcomes of patients. Here, to delineate the driver events of genomic and transcription alteration during HCC recurrence, we performed RNA-Seq profiling of the paired primary and recurrent tumors from two patients with intrahepatic HCC. By comparing the mutational and transcriptomic profiles, we identified somatic mutations acquired by HCC recurrence including novel mutants of *GOLGB1* (E2721V) and *SF3B3* (H804Y). By performing experimental evaluation using siRNA-mediated knockdown and overexpression constructs, we demonstrated that the mutants of *GOLGB1* and *SF3B3* can promote cell proliferation, colony formation, migration, and invasion of liver cancer cells. Transcriptome analysis also revealed that the recurrent HCCs reprogram their transcriptomes to acquire aggressive phenotypes. Network analysis revealed *CXCL8* (*IL-8*) and *SOX4* as common downstream targets of the mutants. In conclusion, we suggest that the mutations of *GOLGB1* and *SF3B3* are potential key drivers for the acquisition of an aggressive phenotype in recurrent HCC.

## INTRODUCTION

Hepatocellular carcinoma (HCC) is the fifth most malignant cancer and the second leading cause of cancer death in the world [1]. Currently, definitive treatment of HCC is surgical resection and liver transplantation [2]. However, tumor recurrence even after curative surgical resection occurs at a rate of 40 % cases within 5 years, resulting in dismal outcomes of HCC patients [3]. Recurrent tumors have been addressed to harbor distinct genomic profiles compared to primary tumors, suggesting an intracellular reprogramming during tumor recurrence. Indeed, recurrent tumors acquire aggressive phenotypes such as invasion, metastasis, epithelial-to-mesenchymal transition (EMT), and chemo-resistance properties, provoking worse prognostic outcomes of tumor patients [4–8]. Therefore, delineating the underlying mechanisms of HCC recurrence are urgently needed to improve clinical outcomes of HCC patients.

Recent explorative advance of high-throughput sequencing technology has allowed us to get overviewed landscapes of genomic variations in cancers, which have revealed genetic drivers that might play critical roles in cancer progression. In HCC, several studies have shown recurrent mutations at *TP53, CTNNB1*, *AXIN1*, *IRF2, CDKN1A*, *CDKN2A*, *ARID1A*, *ARID2*, and *TERT*, which have been addressed to promote HCC development and/or progression implicating clinical outcomes [9–14]. Recurrent tumors showed distinct mutational and/or transcriptomic profiles compared to those of primary tumors [15–17]. These studies imply that the tumor relapse could be derived from the genetic events causing transcriptomic reprogramming of tumor cells. For example, phylogenetic analysis has shown a clonal evolution of the heterogeneous cancer cells with specific mutations can give rise to metastatic or aggressive tumors [18]. Thus, comparison of the genomic and/or transcriptome profiles of the recurrent or metastatic tumors with primary tumors from the same patient might be advantageous in delineating the drivers for recurrent tumor progression.

With this concern, in the present study, we performed an RNA-Seq profiling in the paired primary and recurrent tumors from two patients with intrahepatic HCC (Patient 1 and Patient 2). By comparing the mutation profiles, we identified seven mutations which were found in the recurrent HCCs but not in the primary tumors from the same patients. Of these, we demonstrated that the novel mutants of *GOLGB1* and *SF3B3* can promote HCC progression, providing new mechanistic insights on the HCC relapse.

## RESULTS

### Profiling of RNA-Seq identifies the mutants acquired by recurrence of HCC

**Figure 1:**
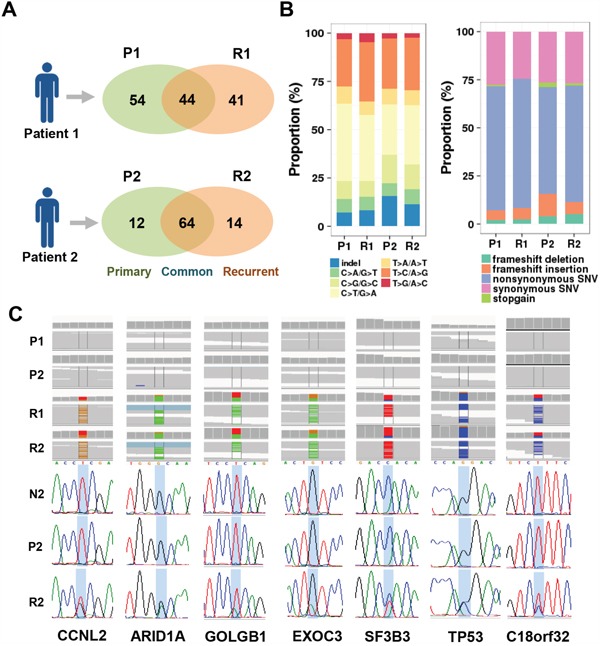
Identification of the mutants-acquired by HCC recurrence **A**. A Venn-diagram shows the number of mutants found in the primary (P1 and P2) and recurrent tumors (R1 and R2) from each of patient 1 and patient 2. **B**. Distribution of the mutation types according to substitution types (*left*) and protein functions (*right*) in each HCC sample is shown. **C**. Assembled sequence leads with mutations are shown by Integrated Genome Viewer (IGV). The seven mutations of common recur-specific mutations were validated by Sanger sequencing method in each of the non-tumoral (N2), primary (P2), and recurrent tumor (R2) tissues from patient 2.

RNA-Seq profiling was performed on the paired intrahepatic primary and recurred tumor specimens from two HCC patients as described in Materials and Methods. The patient 1 had 98 mutations in the primary tumor (P1) and 85 mutations in the recurrent tumor (R1). The patient 1 had 41 recur-specific mutations showing lower mutation retention rate (31.65%). While, the patient 2 had 76 mutations in the primary tumor (P2) and 78 mutations in the recurrent tumor (R2), respectively. The R2 tumor showed higher mutation retention rate (71.11%), having 14 newly acquired recur-specific mutations (Figure 1A). Each tumor had similar mutational spectrum in consistent with previous studies [9, 12]. No significant difference of the mutation spectrum was found between the primary and the recurrent tumors (Figure 1B). Overall, the tumors had frequent mutations of C>T/G>A (38.79%, n=64) and T>C/A>G (22.42%, n=37) (Figure 1B, left). The ratios of nonsynonymous *vs*. synonymous SNVs were ranged from 2.1 to 2.71 (Figure 1B, right).

Among the mutations acquired by HCC recurrence (R1; n=41, R2; n=14), we identified 9 recur-specific mutations which were commonly found in the recurrent tumors but not in the primary and the non-tumoral surrounding tissues. These recur-specific mutations were verified by Sanger sequencing method, but *VHL* (E148fs) and *BAAT* (R297H) were failed to validate. The validated seven recur-specific mutations resided at the genes of *TP53* (P278R), *CCNL2* (R499R), *ARID1A* (G2012D), *GOLGB1* (E2721V), *EXOC3* (V202I), *SF3B3* (H804Y), and *C18orf32* (K75K) (Figure 1C and Table 1). These mutants included previously well-known cancer-associated genes such as *TP53, CCNL2*, and *ARID1A*, implying their functional relevance in the HCC recurrence.

**Table 1:** List of mutations-acquired by HCC recurrence

Genome position	Cytoband	Mutation	Gene	Amino Acid change	PolyPhen2score
Chr1:1322679	1p36.33	T>G	CCNL2	R499R	.
Chr1:27106424	1p36.11	G>A	ARID1A	G2012D	D (score=1)
Chr3:121409809	3q13.33	T>A	GOLGB1	E2721V	D (score=1)
Chr5:453724	5p15.33	G>A	EXOC3	V202I	P (score=0.88)
Chr16:70597900	16q22.1	C>T	SF3B3	H804Y	D (score=0.989)
Chr17:7577105	17p13.1	G>C	TP53	P278R	D (score=1)
Chr18:47008721	18q21.1	T>C	C18orf32	K75K	.

*D: Probably damaging (score >=0.957), P: possibly damaging (0.453<= score <=0.956)

### Transcriptomic reprogramming of the recurrent HCC

**Figure 2:**
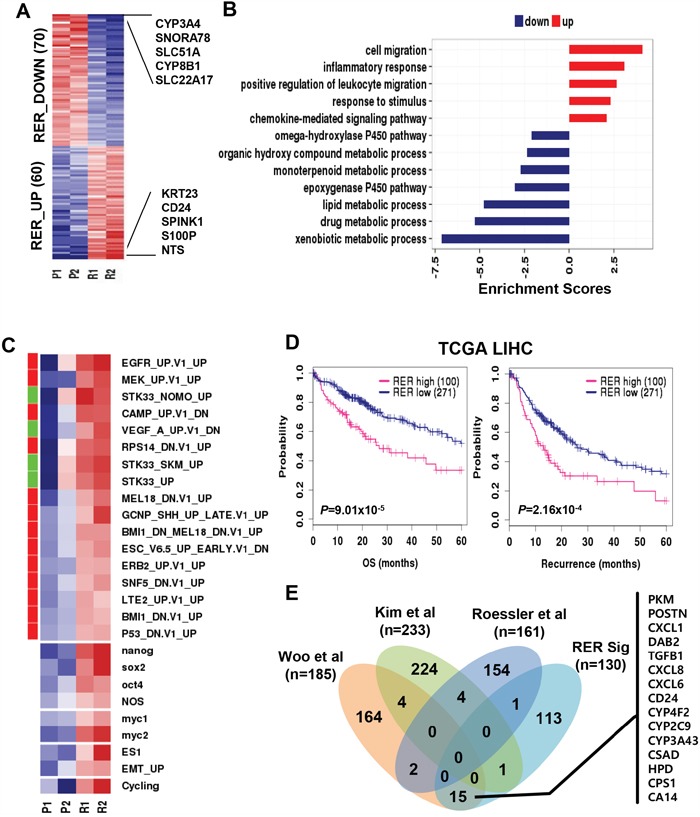
Differential expression between primary and recurrent HCC **A**. A heatmap shows the expression of the gene set of “reprogrammed expression by recurrence (RER)” which are differentially up- and down-regulated genes between the primary and the recurrent HCCs. Top 10-ranked genes with the greatest fold differences are indicated. **B**. A barplot indicates the functional enrichment scores of RER_UP (n=60, *red*) and RER_DOWN (n=70, *blue*) genes which are calculated as described in Materials and Methods. **C**. A heatmap indicates the enrichment scores of the cancer-related gene signatures including the oncogenic features from MsigDB, stemness (ES1, nanog, sox2, oct4, NOS, myc1, and myc2), EMT_UP, and cell cycling in each of HCC samples. The oncogenic (*red*) and tumor suppressive (*green*) features of MSigDB are indicated as a color bar (*left*). **D**. Kaplan-Meier survival curves show the overall survival (*left*) and recurrence-free survival (*right*) between RER_high and RER_low groups in TCGA cohort. **E**. A Venn-diagram shows the number of genes overlapped among the RER_UP genes and the recurrence-related gene signatures from the previous studies of Woo et al., Kim et al., and Rossler et al., respectively.

Next, to delineate concomitant transcriptomic change during HCC relapse, we compared the gene expression levels of the primary and the recurrent HCCs with fold difference greater than two, which revealed 130 differentially expressed genes as the reprogrammed expression by recurrence (RER) including 60 up-regulated (RER_UP) and 70 down-regulated (RER_DOWN) genes (Figure 2A and Supplementary Table 1). Gene ontology (GO) analysis revealed that the RER_UP genes were enriched with the genes related with migration (enrichment scores, ES=4.12) and inflammatory response (ES=3.10), while RER_DOWN genes were enriched with metabolic process-related genes (ES=7.12) (Figure 2B). This result indicates that the RER genes reflected well the aggressive phenotype of the recurrent HCC, implying their regulatory functions on transcriptomic reprogramming during tumor relapse.

To further support the aggressive behavior of the recurrent HCC, we evaluated the expression of the oncogenic signatures from the RNA-seq profile. As expected, the recurrent HCCs demonstrated significant enrichment of oncogenic signatures (*e.g*., *EGFR* and *MEK*) compared to those in the primary HCCs (Figure 2C). Down-regulation of tumor suppressive gene signatures such as TP53_DN.V1_UP, RPS14_DN.V1_UP, and MEL18_DN.V1_UP was observed in the recurrent HCCs. Moreover, consistent with these findings, the recurrent HCCs were enriched with the gene sets which were previously known to associate with cancer aggressiveness, including the gene sets of cell cycling, stemness (ES1, nanog, sox2, oct4, NOS, myc1, and myc2), and EMT-related genes.

In addition, we also evaluated the prognostic relevance of the RER genes in an independent HCC data from TCGA (http://cancergenome.nih.gov). The HCC patients (n=371) were stratified into two groups based on the enrichment scores (ES) of the RER genes which were calculated as described in Materials and Methods. The group with higher RER expression (RER_high, ES > 0, n=100) showed worse prognostic outcomes of overall survival (Hazard ratio HR=2.07, *P*=9.01×10^−5^) and tumor recurrence-free survival (HR=1.87, *P*=2.16×10^−4^) compared to those of the groups with lower RER expression (RER_low, ES < 0, n=271) (Figure 2D). Furthermore, we compared our RER gene signature with the previously reported HCC recurrence-related genes [6–8], but no significant overlap was found among these signatures. This might be due to different use of study design and data platforms. However, the recurrence genes in the previous study *i.e*. Woo et al. [6] had 15 genes overlapped with RER genes, which included many of the well-known representative markers of HCC recurrence such as *CD24* [19], *TGFB1* [20], *CXCL6* [21], *CXCL8* [22], and *PKM* [23] (Figure 2E). These results consistently support that the recurrent HCCs presented more aggressive phenotype at transcriptional level, promoting genomic reprogramming during HCC relapse although limited sample size was used in this study.

### The mutants of *GOLGB1* and *SF3B3* give rise to an aggressive phenotype

**Figure 3:**
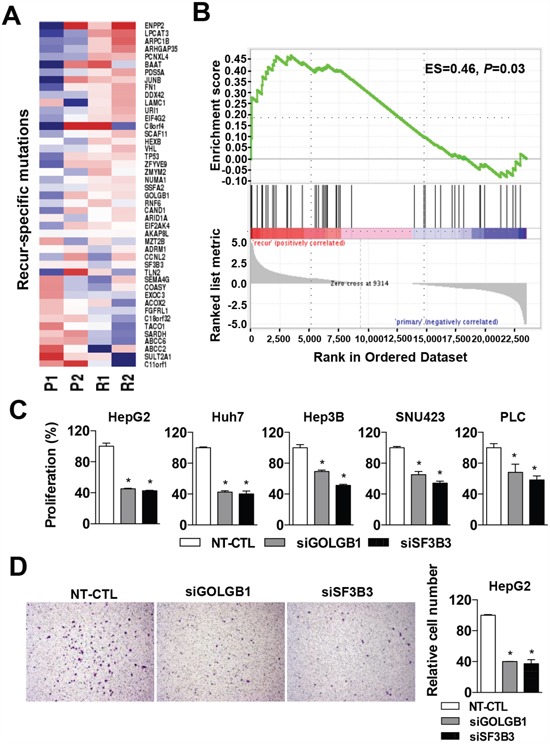
Functional evaluation of the recur-specific mutations **A**. A heatmap indicates the expression levels of recur-specific mutant genes in HCC samples. **B**. Gene set enrichment analysis shows the enriched expression of the recur-specific mutant genes in recurrent HCC compared to those of the primary HCCs. **C**. Effects of the siRNA-mediated knockdown of NT-CTL (non-target control), *GOLGB1* or *SF3B3* for 72 hrs on the cell proliferation are shown in liver cancer cells of HepG2, Huh7, Hep3B, SNU423 and PLC. The cell proliferation activities are determined by a MTT assay. **D**. Cell migration was determined using the Transwell chamber with the cells transfected with siRNAs for NT-CTL (non-target control), *GOLGB1*, and *SF3B3*, respectively. The number of migrated cells for each cell group is quantitated. Data are the mean ± S.D. (*n* = 3). *P < 0.05 with respective control.

Ascertaining the aggressive phenotype of the recurrent HCCs, we next evaluated whether the mutants acquired by recurrence are responsible for the acquisition of the aggressive phenotype in the recurrent HCCs. First, we examined whether the mutations-acquired by recurrence can affect the expression levels of the genes. The expression of the 45 recur-specific mutated genes was significantly enriched in the recurrent HCCs than the primary HCCs (ES=0.46, P-value=0.03, Figure 3A-3B). However, the other mutants excluding the recur-specific mutations showed no significant directional changes of their expression levels between the primary and recurrent HCCs (Supplementary Figure 1). This result may imply that the recur-specific mutations are more likely to act as activating mutations causing overexpression of the mutated genes, although the other mutations might be either activating or inactivating mutations.

Next, to validate the likelihood of activating mutations of the recur-specific mutations, we performed siRNA-mediated knockdown experiments using liver cancer cell lines. Of the seven validated recur-specific mutations (Figure 1B), we focused on the newly identified missense mutants of *GOLGB1* (H804Y) and *SF3B3* (E2721V) because they were predicted to have deleterious structural alterations with Polyphen2 Scores greater than 0.9 (Table 1). Moreover, the functional roles of these genes in HCC progression are largely unknown yet. Indeed, *GOLGB1* mutation was frequently observed in lung cancers (9%), while the *SF3B3* mutation was frequently observed in bladder cancer (5.4%) (Supplementary Figure 2). In HCC, the mutations showed relatively lower frequencies (*GOLGB1*, 2.7%; *SF3B3*, 1.9%).

The siRNA-mediated knockdown of *GOLGB1* and *SF3B3* at transcriptional and protein levels were confirmed by quantitative RT-PCR and western blotting analyses, respectively (Supplementary Figure 3). Both the knockdown cells for *GOLGB1* or *SF3B3* suppressed cell proliferation activity in diverse liver cancer cell lines of HepG2, Huh7, Hep3B, SNU423, and PLC (Figure 3C). Moreover, we also demonstrated that the knockdown of these genes significantly reduced the migration activity of HepG2 cells, indicating their increased metastatic potential (Figure 3D).

**Figure 4:**
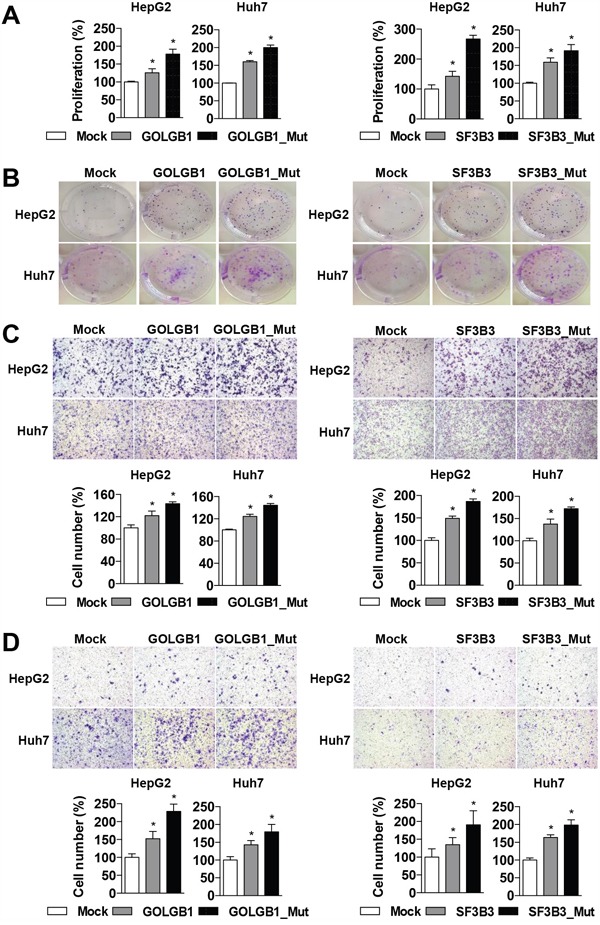
*GOLGB1* and *SF3B3* mutants give rise to aggressive phenotypes **A**. HepG2 and Huh7 Cells are transfected with mock control, wild-type, or mutant-type of *GOLGB1* or *SF3B3* for 72 hrs, respectively, and their effects on cell proliferation are determined by a MTT assay. **B**. Colony formation assays are performed on the cells transfected with mock, wild-type, or mutant-type of *GOLGB1* or *SF3B3* for 14 days, respectively. **C, D**. The HepG2 and Huh7 cells transfected with mock, wild-type, or mutant-type of *GOLGB1* or *SF3B3*, and their effects on cell migration (C) and invasion (D) activities are determined as described in Materials and Methods. The number of migrated or invaded cells is counted and plotted, respectively. Data are the mean ± S.D. (*n* = 3). *P < 0.05 with respective control.

In addition, to evaluate oncogenic functions of the mutants of *GOLGB1* and *SF3B3*, wild-type and mutant-type clones were constructed by performing site-directed mutagenesis experiments. The expression clones were transfected into the liver cancer cells (HepG2 and Huh7), and the overexpression of each form was confirmed at mRNA and protein levels (Supplementary Figure 4). The expression of the wild-type *GOLGB1* or *SF3B3* significantly increased the cell proliferation as well as colony formation ability, respectively. Moreover, the mutant-type forms showed augmenting effects of the wild-type forms, promoting more aggressive cancer phenotypes of cell proliferation, colony formation, migration, and invasion, respectively (Figure 4). These results may indicate that the mutants act as activating mutations. The expression levels of the mutants were not much different between the primary and recurrent HCCs, indicating the mutation effects are not by elevation of the gene expression levels (Supplementary Figure 4).

**Figure 5:**
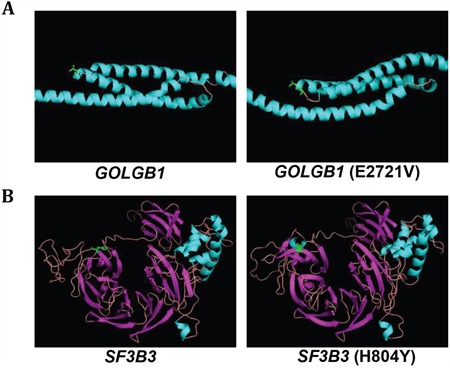
Predicted protein structures for wild-type and mutant-type *GOLGB1* and *SF3B3* **A, B**. The predicted protein structures of the wild-type (*left*) and mutant-type (*right*) of *GOLGB1* (A) and *SF3B3* (B) and are shown. The mutation sites of *GOLGB1* (E2721V) and *SF3B3* (H804Y) are indicated with green color.

The protein structures of the mutants were predicted to have deleterious functional effects by PolyPhen2 software (see Table 1). The mutation of *GOLGB1* (E2721V) was located at a hinged region between α-helix modules, and was predicted to alter the rotation angle of the hinge region (Figure 5A). The mutation of *SF3B3* (H804Y) was located at the boundary of β-sheet and loop structure, and was predicted to alter its loop structure of the wild-type into β-sheet (*i.e*. 805-807 and 813-815 sites) and α-helix (*i.e*. 820-823 sites) structures (Figure 5B). These results support that the mutations may lead to conformational change of proteins structures, promoting aggressive behaviors of cancer cells.

### *CXCL8* and *SOX4* are potential common downstream targets of *GOLGB1* and *SF3B3*

**Figure 6:**
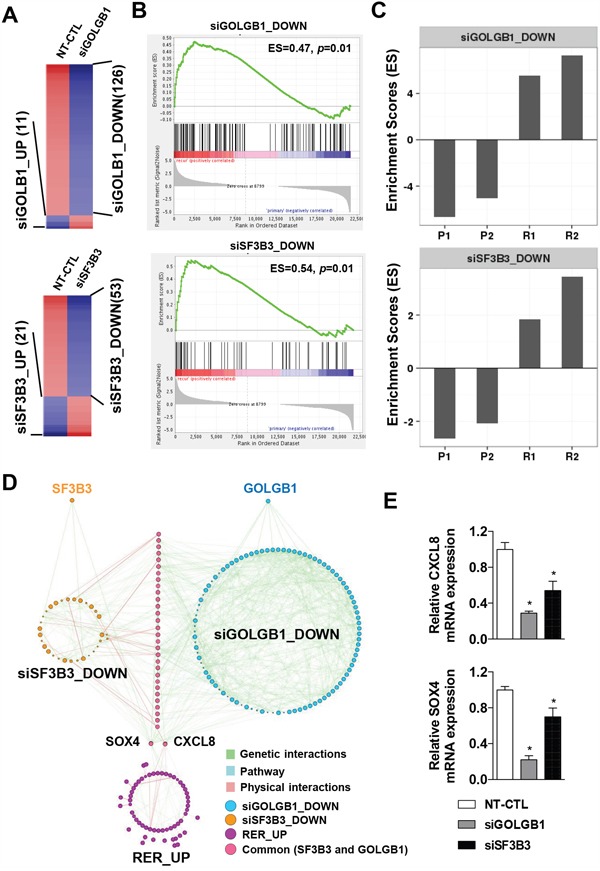
*CXCL8* and *SOX4* are potential downstream targets of the mutants of *GOLGB1* and *SF3B3* **A**. Heatmaps indicate the differentially expressed genes for the siRNA-mediated knockdown HepG2 cells for *GOLGB1* (siGOLGB1, *top*) or *SF3B3* (siSF3B3, *bottom*), respectively. **B**. The enriched expression of the gene sets of siGOLGB1_DOWN (*top*) and siSF3B3_DOWN (*bottom*) between the recurrent and the primary HCCs are shown, respectively. **C**. Bar plots indicate the enrichment scores of the siGOLGB1_DOWN (*top*) and siSF3B3_DOWN (*bottom*) gene signatures in individual HCC samples. **D**. Gene network analysis shows the gene sets of siSF3B3_DOWN (n=53), siGOLGB1_DOWN (n=126), and the RER_UP (n=60) genes with different colors. Gene-to-gene interactions of physical (*blue*), genetic (*green*), and pathways (*red*) are shown with different colors. **E**. The expression levels of *CXCL8* and *SOX4* mRNAs are measured by qRT-PCR in the HepG2 cells transfected with NT-CTL (non-target control), *GOLGB1*, or *SF3B3* siRNAs. Data are the mean ± S.D. (*n* = 3). *P < 0.05 with respective control.

As we demonstrated that the mutations at *GOLGB1* and *SF3B3* can give rise to an aggressive progression in HCC (see Figure 3 and Figure 4), we next sought their potential downstream target genes which might be responsible for the phenotypic alteration by the mutations. By performing gene expression profiling using the knockdown cells for *GOLGB1* or *SF3B3*, we identified the differentially expressed genes (DEG) for *GOLGB1* knockdown (*i.e*. siGOLGB1_UP, n=11, and siGOLGB1_DOWN, n=126) and for *SF3B3* knockdown (*i.e*. siSF3B3_UP, n=21, and siSF3B3_DOWN, n=53), respectively (Figure 6A and Supplementary Table 2). Since the down-regulated genes by the knockdown are thought as their downstream target genes, we evaluated the expression of the siGOLB1_DOWN and siSF3B3_DOWN gene signatures. As expected, we found that the each of the siGOLB1_DOWN and siSF3B3_DOWN gene signatures was significantly enriched in the recurrent HCCs than the primary HCCs (Figure 6B). Individual enrichment scores of these gene signatures also showed marked up-regulation in the recurrent tumors (R1 and R2) compared to the primary tumors (P1 and P2) (Figure 6C). Taken together, we suggest that the genes regulated by *GOLGB1* or *SF3B3* may act as potential downstream effector genes, playing crucial roles in the acquisition of the aggressive phenotype in the recurrent HCCs.

As the siGOLGB1_DOWN and siSF3B3_DOWN signatures are thought as potential targets regulated by *GOLGB1* or *SF3B3*, we next proceeded a network analysis to predict key regulators of the gene expression changes. We constructed gene networks of the siGOLGB1_DOWN, siSF3B3_DOWN, and RER_UP gene signatures, respectively (Figure 6D). The genetic, physical, and pathway interactions among the signature were identified by GeneMANIA software [24]. Both the gene signatures of siGOLGB1_DOWN and siSF3B3_DOWN shared many genes (25.17%, 36 out of 143) each other, implying that the concomitant mutations might have synergic actions together targeting common downstream signaling pathways. With this concern, among these common target genes, we identified that *CXCL8* and *SOX4*, which expression levels were commonly up-regulated in the recurrent HCCs (RER_UP, Figure 6D). The down-regulation of *CXCL8* and *SOX4* expression in the knockdown cells for *GOLGB1* or *SF3B3* were further validated by qRT-PCR (Figure 6E), supporting that *CXCL8* and *SOX4* are the downstream targets of *GOLGB1* and *SF3B3*. Indeed, previous studies have established well that the *CXCL8* (IL-8) and *SOX4* can promote cancer development and progression [25, 26]. In addition, we could observe positive correlations of the expression levels of *GOLGB1* or *SF3B3* with the expression levels of *CXCL8* and *SOX4* in the liver cancer data of TCGA, implying the potential connection between them (Supplementary Figure 5). Taken together, we suggest that the mutations of *GOLGB1* and *SF3B3* act to attain an aggressive phenotype in the recurrent HCC, and which might be potentially through expression of *CXCL8* and *SOX4*.

## DISCUSSION

In the present study, by performing RNA-Seq profiling of the paired primary and recurrent HCCs, we identified seven somatic mutations which were acquired by HCC recurrence. These included well-known cancer genes which mutations were previously found in primary HCCs. For example, *TP53* mutation is one of the most frequent mutation in various cancer types including HCC. The mutated *TP53* promotes aggressive phenotypes such as increased chromosome instability, metastasis, and poor prognosis of patients [27–29]. *ARID1A* is one of key components of chromatin remodeling complex. Frequent mutation of *ARID1A* has been shown in HCC [14, 30, 31], provoking cancer progression and metastasis by epigenetic alteration. *CCNL2* (Cyclin L2) is a member of the cyclin family, which was also reported to have tumor suppressor functions in HCC [32]. Although the sample size was limited in this study, re-identification of these well-known cancer mutants strongly support the reliability of our data and analytic strategy.

Of the recur-specific mutations, to obtain a proof-of- concept, we demonstrated that the novel mutants of *GOLGB1* and *SF3B3* can promote oncogenic features. In fact, oncogenic functions of the *GOLGB1* and *SF3B3* were largely unknown. *GOLGB1*, a golgi apparatus-associated large transmembrane protein [33], has been reported to promote biogenesis and maintenance of compact golgi morphology, but its oncological roles has not been established yet. *SF3B3* is a protein for spliceosome assembly, which expression has been reported to associate with cancer cell growth [34] and prognosis of breast cancer patients [35, 36]. We demonstrated that the overexpression of the *GOLGB1* or *SF3B3* promoted aggressive cancer progression such as cell proliferation, invasion, and migration reflecting aggressive behaviors of the recurrent HCC (see Figure 4). Moreover, the somatic mutations of these genes aggravated the aggressive phenotypes of HCC cells, implying their actions as activating mutations. Structural alteration of the mutant proteins was also predicted supporting their actionability (see Figure 5). Taken together, we suggest that the mutations of *GOLGB1* or *SF3B3* can promote aggressive progression of HCC.

In addition, we identified *CXCL8* and *SOX4* as potential common downstream targets of *GOLGB1* and *SF3B3* (see Figure 6D). *CXCL8* (encoding *IL-8*) activates EGF and MAPK signaling cascades resulting in cancer progression and metastasis in diverse cancer types [37, 38]. *SOX4* is an important regulator of EMT functioning in cancer progression [39]. Knockdown of *SOX4* could suppress HCC cell migration, invasion, and intrahepatic metastasis [40]. Taken together, we suggest that the *CXCL8* and *SOX4* are key down-stream effectors for the aggressive progression by the mutations *GOLGB1* and/or *SF3B3*. However, the mechanistic relationships of *GOLGB1* or *SF3B3* with *CXCL8* and/or *SOX4* were not fully evaluated in this study, requiring further extended investigation.

In conclusion, by performing combined analyses of the mutation and transcriptome profiles and experimental evaluation, we successfully identified novel driver mutations and their potential target genes. Targeting *GOLGB1* or *SF3B3* might have therapeutic or diagnostic advantages in precision management of HCC patients.

## MATERIALS AND METHODS

### Patients and specimens

Surgically resected HCC specimens of the paired primary and recurrent tumors from two HCC patients were obtained from the Ajou Human Bio-Resource Bank (AHBB), a member of the National Biobank of Korea, which is supported by the Ministry of Health and Welfare. Both the recurrent tumors occurred 14 months after surgical resection. The Institutional Review Board of Ajou University Hospital at Republic of Korea has approved this study, and waived the need for informed consent from donors.

### RNA-Seq profiling and data processing

Total RNA was isolated using TRIzol® RNA Isolation Reagents (Life technologies, Carlsbad, CA), and the RNA integrity was confirmed by a bioanalyzer using an Agilent RNA 6000 Pico Kit (Agilent, Santa Clara, CA). The sequencing library for mRNA was constructed using TruSeq RNA sample preparation kit (illumina, San Diego, CA) according to manufacturer's instruction. Sequencing reaction was performed on an illumina HiSeq2000 for 100-bp paired end reads (2 × 100) with coverage greater than 30 million reads per sample. The raw image data was transformed and stored in FASTQ format. The low quality sequence reads with less than 20 PHRED score were masked to ‘N’ using ‘fastq_masker’ command of FASTX-toolkit (http://hannonlab.cshl.edu/fastx_toolkit/), then mapped to human reference genome (hg19) using Tophat [41] with default parameters. The PCR duplicates were removed by ‘picard’ MarkDuplicates (http://picard.sourceforge.net). The gene expression levels from RNA-Seq data were estimated by the log2-transformed FPKM values using Cufflinks [42].

### Variant calling from RNA-Seq data

The RNA-Seq data were processed for variant calling. Local realignment of indel and normalization of base quality scores were performed using GATK IndelRealigner and Recalibrator, respectively [43]. The sequence variations were filtered by using GATK UnitiedGenotyper [43] with following conditions: (i) MQ0<4 and MQ0/(1.0*DP)>0.1, (ii) DP<5, (iii) QUAL<50, (iv) QD<1.5, where MQ0 indicate number of reads in which mapping quality zero, DP indicate read depth on variant position, QUAL is base call quality and QD is variant confidence by depth. In addition, the sequence variations with under 10 mutant read depth, known SNP (dbSNP138), or non-exonic variants were further filtered out, then the variants were annotated by using ANNOVAR software [44]. The identified mutants were validated using Sanger sequencing method.

### Microarray gene expression profiling

Total RNA was amplified and purified using the Ambion Illumina RNA amplification kit (Ambion, Austin, TX) to yield biotinylated complementary DNA (cDNA) according to the manufacturer's instruction. Briefly, 550 ng of total RNA was reverse-transcribed to cDNA using deoxythymidine oligomer primer and labeled with biotinylated deoxyribonucleotide triphosphate. Labeled cDNA samples were hybridized to human HT-12 expression v.4 bead arrays, and the signal intensity was detected according to the manufacturer's instruction (Illumina, San Diego, CA). Raw data were processed by log2 transformation and quantile normalization.

### Gene ontology and gene set enrichment analyses

Gene ontology (GO) analysis of the gene sets was performed using g:Profiler R package [45]. Statistical significance was determined with a cutoff of P < 0.01. To evaluate the aggressive phenotype of the recurrent HCC, we obtained 189 oncogenic signatures from mSigDB [46], and other cancer-related genes including stemness (*i.e*., ES1, nanog, sox2, oct4, NOS, myc1, myc2) [47], EMT [48], and cell cycle-related genes which have been reported previously.

Functional enrichment score for a gene signature was determined by applying Kolmogorov-Smirnov (KS) test. For each sample, directional P-values for the estimates D+ and D- were calculated by KS-test, and the enrichment score for a given signature was calculated as -log10 (P-value) as described previously [49]. In addition, gene set enrichment analysis between sample groups was performed using GSEA software [46]. All the statistical computation was performed using R software (http://www.r-project.org).

### Cell culture and siRNA-mediated knockdown experiments

Human HCC cell lines (*i.e*., HepG2, Huh7, Hep3B, SNU423, and PLC) obtained from the American Type Culture Collection (ATCC, Manassas, VA) were cultured in Dulbecco's modified Eagle's medium (DMEM) supplemented with 10% fetal bovine serum (Gibco BRL, Grand Island, NY) and 100U penicillin/streptomycin at 37°C in a humidified 5% CO2 incubator. Non-targeting control siRNA and siRNAs against human *GOLGB1* and *SF3B3* were purchased from Dharmacon Inc. (Lafayette, CO). Cells were transfected with each siRNA using Lipofectamine 3000 (Invitrogen, Carlsbad, CA) according to the manufacturer's instruction.

### Construction of expression vectors

*GOLGB1* and *SF3B3* constructs were cloned by using In-fusion cloning method (Clontech, Mountain View, CA). Briefly, wild- and mutant-type clones for *GOLGB1* or *SF3B3* were constructed by CloneAmp HiFi PCR premix (TAKARA, Tokyo, Japan) with specific primers (Supplementary Table 3). Large sized *GOLGB1* was constructed by two bricks strategy using head (1 ~ 5029 bp) and tail (5030 ~ 9810 bp) bricks. The cDNA fragments were inserted into pcDNA3 vector using EcoRI and XbaI. The PCR products of the mutants confirmed by Sanger DNA sequencing method.

### Cell proliferation and colony formation assays

2×10^3^ cells per well were seeded in 96-well plates and incubated at 37°C in a humidified incubator containing 5% CO_2_ overnight. After transfection for indicated time period, 5 mg/mL MTT solution (Amresco, Cleveland, OH) was added to each well and incubated for 2 h. The blue crystalline precipitate in each well was dissolved in 150 μl per well DMSO, and the visible absorbance at 550 nm of each well was quantified using a microplate reader. All the experiments were performed in triplicate. For colony formation assay, cells were transfected with the constructs of mock control, and wild- or mutant-types of *GOLGB1* or *SF3B3* for 48 h, respectively. Then, 500 cells/well were seeded in the 6-well plates for 14 days. The colonies were washed 2 times with phosphate-buffered saline (PBS), fixed with 3.7% Paraformaldehyde, and stained with 1% crystal violet solution in distilled water.

### Cell migration and invasion assays

Cell migration and invasion assays were performed in 8-μm-pore Transwells (6.5 mm; Costar, Corning, NY) with the Transwell filters of uncoated (for migration) or re-coated (for invasion) with matrigel (100 μl, diluted 1:10 in PBS for 1h, BD Biosciences, Bedford, MA), respectively. After rinsing the filters with PBS, the cells were plated and incubated for indicated time periods at 37°C, 5% CO2. Non-invaded or migrated cells in the upper chamber were removed with a cotton swab. The invaded or migrated cells through the filter were washed with PBS, fixed with 3.7% formalin, and stained with 0.1% crystal violet for 1 h. The number of invaded or migrated cells was counted under a light microscope.

### Quantitative real time-PCR (qRT-PCR)

Total RNA was extracted from cells using the RNAiso plus (Takara, Tokyo, Japan). cDNA was synthesized from 2 μg of total RNA using PrimeScript RT reagent Kit (Perfect Real Time) (Takara, Tokyo, Japan). Quantitation of the gene expression levels were performed by a CF96TM Optics Module using IQ SYBR Super Mix (Bio-Rad, Richmond, CA) with the specific primers (Supplementary Table 3). All reactions were triplicated, and the 2^−ΔΔ^Ct values were used for quantification.

### Western blotting

Cells were washed twice with PBS and lysed with RIPA lysis buffer containing protease and phosphatase inhibitors at 4°C for 30 min. 20 μg of total protein was loaded onto 8% SDS-polyacrylamide gel electrophoresis (PAGE) and transferred onto nitrocellulose membranes (Bio-Rad). Membranes were blocked for 1 h in 0.2% I-Block™ (Applied Biosysms) in TBST and then incubated for 1 h at room temperature or at 4°C overnight with primary antibody diluted in TBST containing 0.2% I-Block™. The primary antibodies for anti-*GOLGB1* (R&D Systems, Minneapolis, MN) and anti-*SF3B3* (Santa Cruz Biotechnology, Dallas, TX) were used. After three washes with TBST, the blots were incubated for 1 h with horseradish peroxidase-conjugated secondary anti-mouse (AbFrontier, Seoul, Korea) or anti-sheep (Abcam, Cambridge, MA) antibodies. The immunoblots were visualized by EzWestLumi (Atto, Tokyo, Japan).

### Protein structure analysis

Protein structures of wild- and mutant-types of *GOLGB1* or *SF3B3* were predicted using PSIPRED [50] with default parameters. The best scored protein model was selected, and its 3D structure was visualized using PyMOL (http://www.pymol.org).

## SUPPLEMENTARY FIGURES AND TABLES





## Abbreviations

HCC: hepatocellular carcinoma
EMT: epithelial-to-mesenchymal transition
FPKM: Fragments Per Kilobase Million
GO: Gene ontology
KS: Kolmogorov-Smirnov
RER: reprogrammed expression by recurrence
ES: enrichment score
TCGA: The Cancer Genome Atlas
DEG: differentially expressed gene
IGV: Integrated Genome Viewer
