# Supplementary material for: Mutations acquired by hepatocellular carcinoma recurrence give rise to an aggressive phenotype
Source: Oncotarget. 2016 Dec 27;8(14):22903–16. doi: 10.18632/oncotarget.14248 (PMC5410272; doi:10.18632/oncotarget.14248)
Supplement: Supplementary file 1 [file oncotarget-08-22903-s001.pdf]

Mutations acquired by hepatocellular carcinoma recurrence give rise to an aggressive phenotype

SUPPLEMENTARY FIGURES AND TABLES

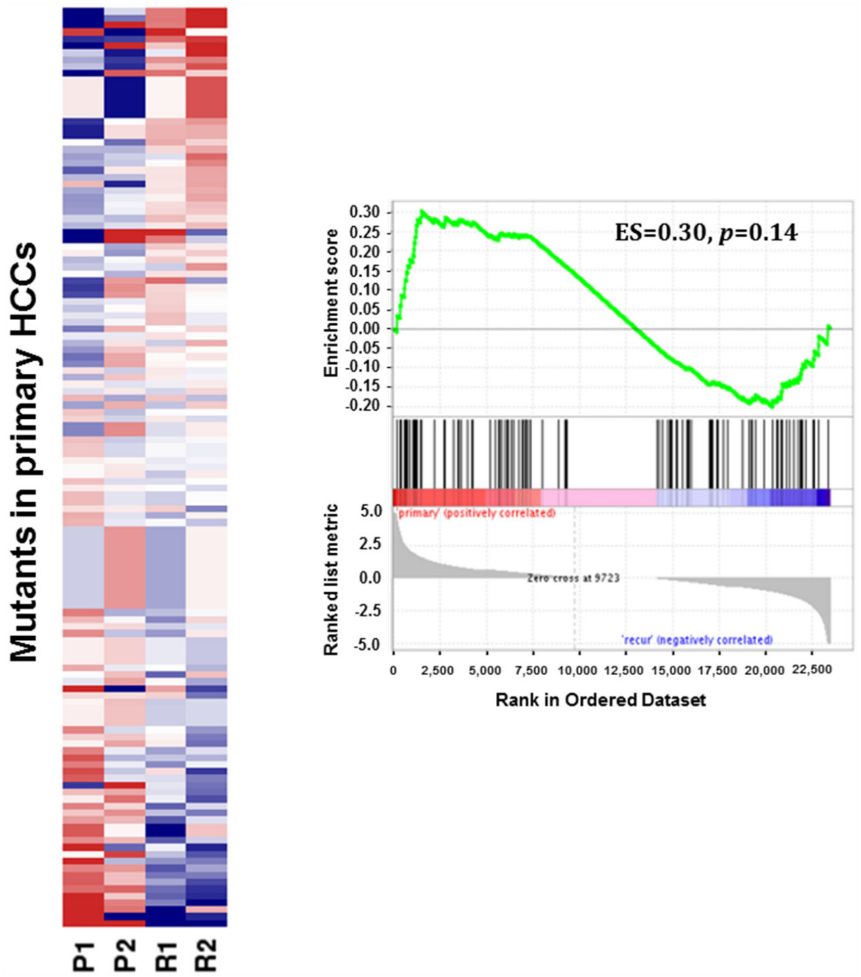

Supplementary Figure 1: Gene expression profiles and gene set enrichment analysis of the mutants in the primary HCCs. A heatmap indicates gene expression levels of the mutants found in the primary (P1 and P2) but not recurrent HCCs (R1 and R2) (*left*). Gene set enrichment analysis shows no significant enrichment of the mutated genes in the primary tumors between the primary and recurrent HCCs (*right*).

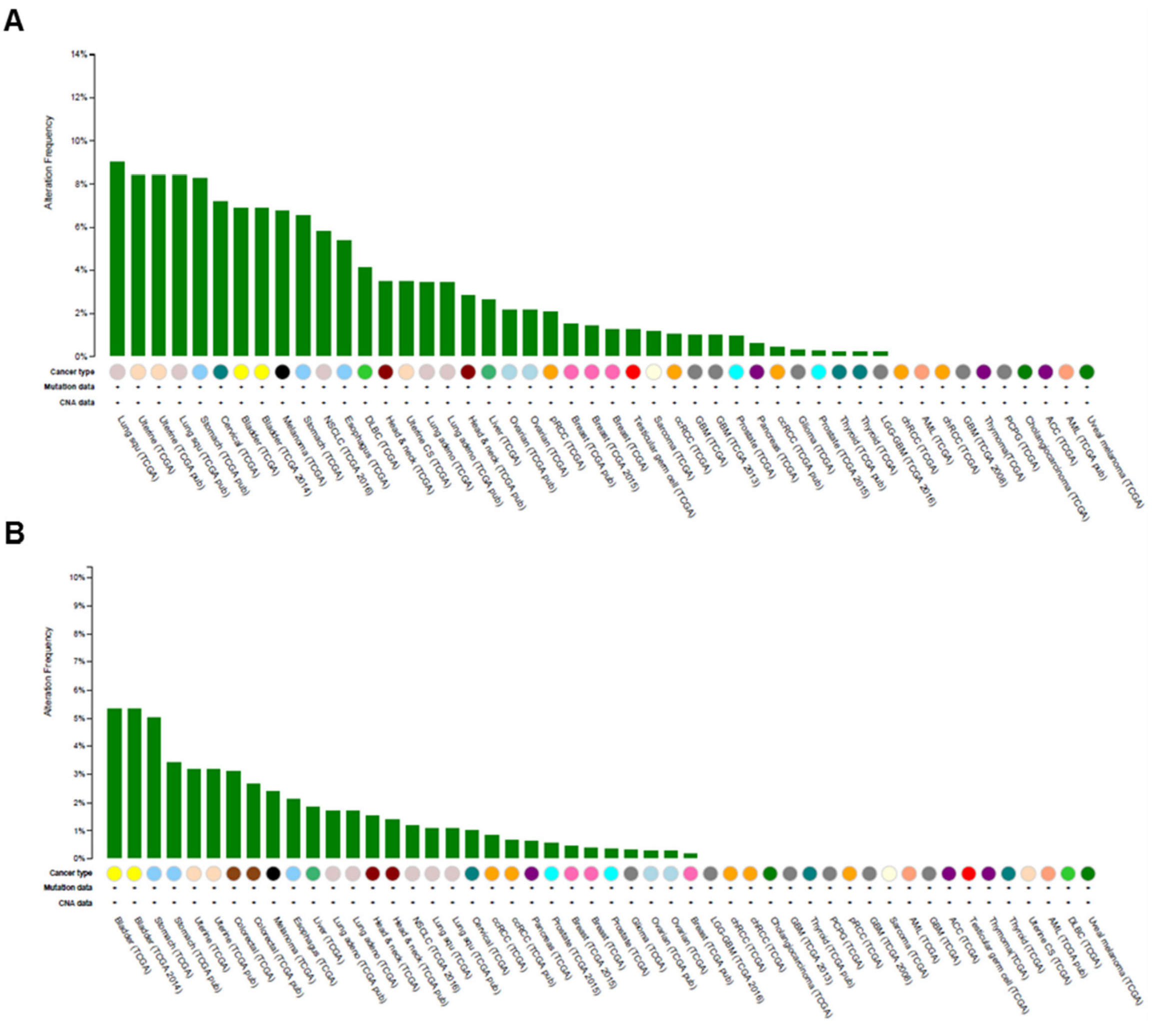

Supplementary Figure 2: The mutation frequency of *GOLGB1* and *SF3B3* genes from TCGA data. The bar plots show the mutation frequency of *GOLGB1* **A.** and *SF3B3* **B.** genes in various cancer types from cBioPortal database (http://www.cbioportal.org/).

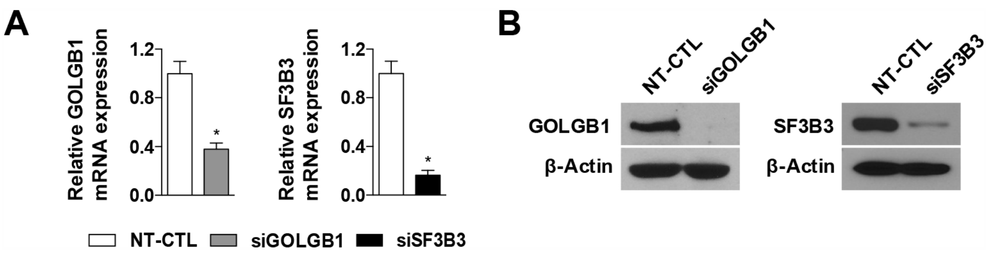

Supplementary Figure 3: The expression levels of *GOLGB1* and *SF3B3* mRNAs and proteins in the siRNA-mediated knockdown cells. A. The expression levels of *GOLGB1* or *SF3B3* mRNAs are measured by qRT-PCR in the HepG2 cells transfected with NT-CTL (non-target control), *GOLGB1* or *SF3B3* siRNA for 24 h. Data indicate the mean ± S.D. ($n = 3$). *$P < 0.05$ with respective control. **B.** The expression levels of *GOLGB1* or *SF3B3* proteins were measured by western blot analysis in the HepG2 transfected with NT-CTL (non-target control), *GOLGB1* or *SF3B3* siRNA for 48 h.

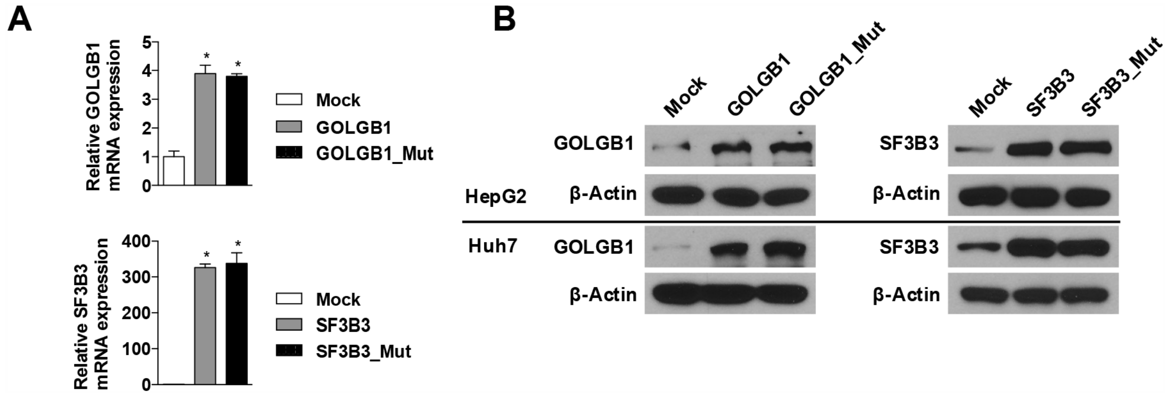

Supplementary Figure 4: The expression of *GOLGB1* and *SF3B3* mRNA and protein levels in the overexpressed cells for the wild- and mutant-types of *GOLGB1* or *SF3B3*. A. The wild- and mutant types expression levels of *GOLGB1* or *SF3B3* mRNAs are measured by real-time qPCR in the HepG2 cells transfected with mock as a control, wild- or mutant forms of *GOLGB1* or *SF3B3* for 24 h. Data indicate the mean ± S.D. ($n = 3$). $*P < 0.05$ with respective control. **B.** The wild- and mutant-form expression levels of *GOLGB1* or *SF3B3* proteins are measured by western blot analysis in the HepG2 and Huh7 transfected with mock as a control, normal or mutated forms of *GOLGB1* or *SF3B3* for 48 h.

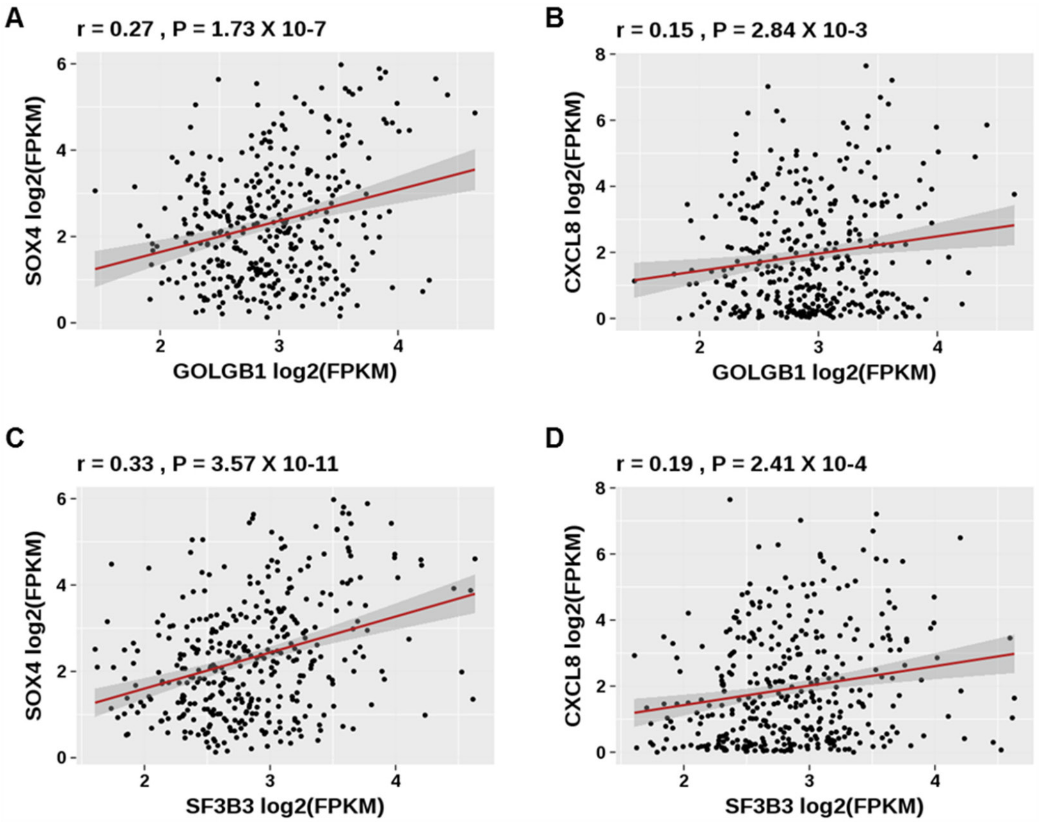

Supplementary Figure 5: Correlation of the expression levels of *SF3B3*/*GOLGB1* and *SOX4*/*CXCL8*. A-D. Correlations between the expression levels of *GOLGB1* (A, B) or *SF3B3* (C, D) with the expression levels of *SOX4* and *CXCL8* are shown, respectively. The gene expression data are obtained from intrahepatic liver cancer data in TCGA data portal (http://cancergenome.nih.gov).

Supplementary Table 1: Gene list of RER signature

See Supplementary File 1

Supplementary Table 2: Gene lists of the siRNA-mediated knockdown signatures for *GOLGB1* or *SF3B3*

See Supplementary File 1

Supplementary Table 3: List of primers for qRT-PCR and sequencing reactions

See Supplementary File 1
