# Supplementary material for: Mutations acquired by hepatocellular carcinoma recurrence give rise to an aggressive phenotype
Source: Oncotarget. 2016 Dec 27;8(14):22903–16. doi: 10.18632/oncotarget.14248 (PMC5410272; doi:10.18632/oncotarget.14248)
Supplement: Supplementary file 2 [file oncotarget-08-22903-s002.docx]

Supplementary Tables

**Supplementary Table S1. Gene list of RER signature**

| **RER_UP** | **FOLD DIFFERENCE**  **(R1 *vs.* P1)** | **FOLD DIFFERENCE (R2 *vs.* P2)** |
| --- | --- | --- |
| NTS | 5.504 | 8.084 |
| S100P | 5.803 | 7.342 |
| SPINK1 | 6.616 | 4.643 |
| CD24 | 3.639 | 6.236 |
| KRT23 | 3.852 | 5.412 |
| TMPRSS15 | 4.383 | 4.306 |
| LCN2 | 3.078 | 5.533 |
| CXCL1 | 4.693 | 3.797 |
| CXCL6 | 3.313 | 4.895 |
| SNORA16A | 4.102 | 4.026 |
| SNORA64 | 4.102 | 4.026 |
| AGR2 | 2.542 | 5.557 |
| VNN2 | 3.945 | 3.968 |
| CTHRC1 | 3.219 | 4.409 |
| LGALS7B | 2.202 | 5.304 |
| CCL20 | 5.405 | 2.096 |
| CXCL8 | 4.160 | 3.111 |
| LY96 | 4.951 | 2.249 |
| MSC | 2.762 | 4.188 |
| MAGEA1 | 3.106 | 3.626 |
| SIPA1L2 | 2.552 | 4.100 |
| LGALS3 | 3.754 | 2.760 |
| TRNP1 | 3.535 | 2.948 |
| RAB3C | 3.747 | 2.672 |
| LYZ | 3.398 | 2.919 |
| TIMP1 | 4.036 | 2.277 |
| MMP7 | 2.957 | 3.245 |
| ARSF | 3.787 | 2.410 |
| TMC5 | 2.542 | 3.540 |
| TSPAN13 | 2.526 | 3.390 |
| GBP2 | 3.082 | 2.791 |
| CYP4F22 | 3.252 | 2.557 |
| DAB2 | 3.305 | 2.467 |
| BAALC | 2.107 | 3.613 |
| CXCL17 | 2.207 | 3.475 |
| SULF1 | 2.731 | 2.930 |
| SOX4 | 3.221 | 2.299 |
| TUBB2A | 2.864 | 2.604 |
| CXCL5 | 2.072 | 3.333 |
| DUOX2 | 2.919 | 2.474 |
| BTNL8 | 2.402 | 2.723 |
| TUBB2B | 2.651 | 2.452 |
| CDS1 | 2.344 | 2.733 |
| ADAMDEC1 | 2.263 | 2.772 |
| SELM | 2.140 | 2.857 |
| GPX2 | 2.161 | 2.812 |
| TESC | 2.687 | 2.274 |
| TMEM156 | 2.244 | 2.708 |
| VNN1 | 2.767 | 2.172 |
| CD109 | 2.068 | 2.847 |
| POSTN | 2.393 | 2.497 |
| MARCKSL1 | 2.550 | 2.299 |
| CCDC109B | 2.671 | 2.126 |
| PKM | 2.240 | 2.436 |
| SLC1A5 | 2.119 | 2.513 |
| APOBEC3B | 2.461 | 2.033 |
| BMP2 | 2.317 | 2.054 |
| A2M | 2.221 | 2.062 |
| FGF13 | 2.102 | 2.159 |
| TGFB1 | 2.166 | 2.017 |
| **RER_DOWN** | **FOLD DIFFERENCE**  **(R1 *vs.* P1)** | **FOLD DIFFERENCE**  **(R2 *vs.* P2)** |
| CYP3A4 | -7.929 | -5.753 |
| SNORA78 | -4.720 | -4.943 |
| SLC51A | -4.562 | -4.655 |
| CYP8B1 | -3.413 | -5.506 |
| SLC22A17 | -2.331 | -6.461 |
| EEF1A2 | -4.220 | -4.416 |
| LCAT | -4.429 | -4.021 |
| CNDP1 | -3.346 | -5.009 |
| ADH4 | -2.745 | -5.589 |
| SNORA5A | -4.050 | -4.233 |
| SNORD10 | -3.942 | -4.121 |
| CA14 | -4.737 | -3.291 |
| CRYAA | -3.582 | -4.279 |
| NAT2 | -2.900 | -4.911 |
| SNORA75 | -3.815 | -3.982 |
| HOGA1 | -3.874 | -3.758 |
| CYP1A2 | -4.958 | -2.602 |
| CLRN3 | -2.066 | -5.486 |
| TTC36 | -2.350 | -5.197 |
| ACADL | -3.618 | -3.636 |
| THRSP | -2.447 | -4.697 |
| HPD | -2.310 | -4.607 |
| NEU4 | -4.337 | -2.567 |
| MOGAT2 | -2.926 | -3.872 |
| CYP3A43 | -3.416 | -3.352 |
| PCK1 | -2.405 | -4.354 |
| BSCL2 | -3.035 | -3.686 |
| DNAJC12 | -3.821 | -2.833 |
| PRSS8 | -3.563 | -3.055 |
| CPS1 | -2.600 | -3.903 |
| GREM2 | -2.311 | -4.009 |
| CYP4F2 | -2.043 | -4.224 |
| KRT222 | -2.371 | -3.878 |
| GADD45G | -3.688 | -2.526 |
| PYROXD2 | -3.439 | -2.761 |
| CHRNA4 | -3.154 | -2.913 |
| CYP2C9 | -2.755 | -3.215 |
| SLC1A2 | -3.589 | -2.310 |
| IL27 | -3.516 | -2.369 |
| MACROD1 | -3.061 | -2.711 |
| APOA5 | -2.224 | -3.534 |
| UBXN10 | -2.340 | -3.280 |
| SUSD3 | -3.160 | -2.434 |
| KRTCAP3 | -2.704 | -2.843 |
| GLYAT | -2.065 | -3.420 |
| CLDN14 | -3.321 | -2.140 |
| MME | -3.074 | -2.296 |
| TERT | -2.721 | -2.627 |
| CYP2B6 | -2.763 | -2.518 |
| SLX1B | -2.987 | -2.216 |
| METRN | -2.745 | -2.356 |
| OLFML1 | -2.060 | -3.032 |
| PBLD | -2.874 | -2.202 |
| RBP5 | -2.184 | -2.874 |
| PPP1R1A | -2.915 | -2.127 |
| CIDEB | -2.387 | -2.569 |
| ZDHHC11 | -2.625 | -2.204 |
| SIK1 | -2.641 | -2.025 |
| SLC6A13 | -2.502 | -2.113 |
| SULT1A2 | -2.483 | -2.119 |
| CSAD | -2.569 | -2.009 |
| TMEM100 | -2.301 | -2.270 |
| PANK1 | -2.436 | -2.117 |
| RCAN1 | -2.218 | -2.297 |
| LIME1 | -2.341 | -2.159 |
| ZMYND12 | -2.379 | -2.077 |
| TMEM25 | -2.210 | -2.242 |
| TM6SF2 | -2.199 | -2.209 |
| CES3 | -2.165 | -2.167 |
| CRYM | -2.077 | -2.096 |

**Supplementary Table S2. Gene lists of the siRNA-mediated knockdown signatures for *GOLGB1* or *SF3B3***

| **siGOLGB1_DOWN** | **FOLD DIFFERENCE**  **(siGOLGB1 *vs.* NT-CTL)** | **siGOLGB1_UP** | **FOLD DIFFERENCE**  **(siGOLGB1 *vs.* NT-CTL)** |
| --- | --- | --- | --- |
| SERPINE1 | -2.378 | LAMC1 | 1.596 |
| CXCL8 | -2.234 | CDK6 | 1.540 |
| RASD1 | -1.989 | RABL3 | 1.334 |
| SYT11 | -1.845 | GINS2 | 1.295 |
| ITGA2 | -1.840 | C16orf75 | 1.293 |
| GOLGB1 | -1.834 | CDK2 | 1.260 |
| GDF15 | -1.797 | IMPA2 | 1.055 |
| IGFBP1 | -1.680 | RNF144 | 1.042 |
| THUMPD1 | -1.653 | FZD4 | 1.029 |
| DUSP5 | -1.605 | SNRPB2 | 1.021 |
| PLAU | -1.595 | RXRA | 1.002 |
| ESAM | -1.584 |  |  |
| RAB5A | -1.549 |  |  |
| F2RL1 | -1.509 |  |  |
| CPXM1 | -1.467 |  |  |
| ACTA1 | -1.467 |  |  |
| ARL1 | -1.463 |  |  |
| PALLD | -1.451 |  |  |
| COMMD2 | -1.445 |  |  |
| TUBB2B | -1.438 |  |  |
| SOX4 | -1.434 |  |  |
| RGL1 | -1.417 |  |  |
| MCL1 | -1.409 |  |  |
| BNIP2 | -1.400 |  |  |
| S100A3 | -1.364 |  |  |
| IER3 | -1.361 |  |  |
| C8orf4 | -1.355 |  |  |
| SSR1 | -1.338 |  |  |
| EHBP1 | -1.338 |  |  |
| ANO6 | -1.323 |  |  |
| MARCKS | -1.322 |  |  |
| COQ10B | -1.321 |  |  |
| TNFRSF12A | -1.316 |  |  |
| NOS3 | -1.304 |  |  |
| NUAK1 | -1.300 |  |  |
| FZD6 | -1.287 |  |  |
| RSPRY1 | -1.283 |  |  |
| EVA1A | -1.279 |  |  |
| GBP2 | -1.278 |  |  |
| CUTC | -1.270 |  |  |
| JKAMP | -1.267 |  |  |
| PLIN5 | -1.258 |  |  |
| NDRG1 | -1.250 |  |  |
| FAM43A | -1.248 |  |  |
| WDR26 | -1.229 |  |  |
| FST | -1.224 |  |  |
| CYP3A5 | -1.222 |  |  |
| STK3 | -1.222 |  |  |
| IMPA1 | -1.220 |  |  |
| ZNF280A | -1.215 |  |  |
| RB1CC1 | -1.212 |  |  |
| GMFB | -1.208 |  |  |
| GCNT3 | -1.207 |  |  |
| ZFP36 | -1.204 |  |  |
| MXD1 | -1.203 |  |  |
| CCNJ | -1.197 |  |  |
| ABRACL | -1.190 |  |  |
| ARG2 | -1.183 |  |  |
| LONRF1 | -1.181 |  |  |
| NECAP1 | -1.178 |  |  |
| EIF4EBP2 | -1.167 |  |  |
| C1orf64 | -1.163 |  |  |
| CAMK2N1 | -1.155 |  |  |
| FAM175A | -1.146 |  |  |
| ENPP1 | -1.146 |  |  |
| CHIC2 | -1.144 |  |  |
| FHL3 | -1.140 |  |  |
| SACM1L | -1.115 |  |  |
| ACVR1 | -1.113 |  |  |
| HBEGF | -1.112 |  |  |
| SLC30A7 | -1.109 |  |  |
| STK39 | -1.107 |  |  |
| ANKRD1 | -1.097 |  |  |
| MMD | -1.097 |  |  |
| STAM | -1.097 |  |  |
| CEMIP | -1.096 |  |  |
| INPP1 | -1.093 |  |  |
| ANKRD46 | -1.090 |  |  |
| SATB2 | -1.081 |  |  |
| KRT19 | -1.079 |  |  |
| STK26 | -1.076 |  |  |
| ZFAND5 | -1.075 |  |  |
| COL7A1 | -1.075 |  |  |
| FAM3C | -1.075 |  |  |
| CALM1 | -1.073 |  |  |
| MOSPD1 | -1.072 |  |  |
| PHLDA2 | -1.070 |  |  |
| RCL1 | -1.070 |  |  |
| CDR2L | -1.070 |  |  |
| MBNL2 | -1.069 |  |  |
| DNAJB9 | -1.068 |  |  |
| ATP1B1 | -1.067 |  |  |
| KLHL2 | -1.066 |  |  |
| TMEM199 | -1.064 |  |  |
| TMEM181 | -1.062 |  |  |
| PGM2 | -1.057 |  |  |
| NES | -1.057 |  |  |
| ERO1A | -1.056 |  |  |
| TUBB2A | -1.055 |  |  |
| SPOCK2 | -1.055 |  |  |
| STARD3NL | -1.052 |  |  |
| NCOA7 | -1.047 |  |  |
| EGR1 | -1.044 |  |  |
| TRNP1 | -1.038 |  |  |
| FAM104A | -1.037 |  |  |
| WSB2 | -1.035 |  |  |
| TRIB1 | -1.032 |  |  |
| ZFP36L1 | -1.031 |  |  |
| RAD23A | -1.030 |  |  |
| GLIPR1 | -1.029 |  |  |
| CD3D | -1.025 |  |  |
| MET | -1.024 |  |  |
| GK | -1.024 |  |  |
| TTL | -1.023 |  |  |
| GRAMD1A | -1.023 |  |  |
| DBNDD2 | -1.021 |  |  |
| ANXA3 | -1.020 |  |  |
| SLC35G2 | -1.020 |  |  |
| GTF2E1 | -1.017 |  |  |
| TDG | -1.016 |  |  |
| DFNA5 | -1.016 |  |  |
| CERS6 | -1.013 |  |  |
| CNOT8 | -1.010 |  |  |
| RC3H2 | -1.007 |  |  |
| SCYL2 | -1.003 |  |  |
| SLC2A6 | -1.003 |  |  |
| **siSF3B3_DOWN** | **FOLD DIFFERENCE**  **(siSF3B3 *vs.* NT-CTL)** | **siSF3B3_UP** | **FOLD DIFFERENCE**  **(siSF3B3 *vs.* NT-CTL)** |
| SF3B3 | -1.768 | LAMC1 | 2.020 |
| SYT11 | -1.679 | CDK6 | 1.942 |
| IL8 | -1.671 | RNF144 | 1.524 |
| GDF15 | -1.671 | C16orf75 | 1.409 |
| RASD1 | -1.577 | GINS2 | 1.405 |
| ITGA2 | -1.479 | HAMP | 1.358 |
| HSPE1 | -1.470 | MTMR6 | 1.172 |
| DUSP5 | -1.382 | MKRN1 | 1.166 |
| RWDD4A | -1.360 | CLDN12 | 1.151 |
| SCML2 | -1.336 | DCAF16 | 1.148 |
| ESAM | -1.331 | RABL3 | 1.133 |
| DFNA5 | -1.331 | CDK2 | 1.128 |
| DDAH1 | -1.302 | PRMT3 | 1.107 |
| IER3 | -1.298 | LOC729200 | 1.080 |
| GMFB | -1.257 | C11orf75 | 1.064 |
| SOX4 | -1.251 | GEMIN5 | 1.061 |
| ACTA1 | -1.245 | PPP3R1 | 1.039 |
| PLAU | -1.242 | INTS5 | 1.038 |
| HDDC2 | -1.235 | FZD4 | 1.025 |
| FAM176A | -1.232 | SNRPB2 | 1.019 |
| FERMT2 | -1.214 | IMPA2 | 1.013 |
| IGFBP1 | -1.210 |  |  |
| MCL1 | -1.209 |  |  |
| ORMDL3 | -1.203 |  |  |
| S100A3 | -1.194 |  |  |
| ALDH9A1 | -1.174 |  |  |
| FAM3C | -1.163 |  |  |
| TNFRSF12A | -1.158 |  |  |
| WWC1 | -1.149 |  |  |
| CCL20 | -1.145 |  |  |
| TMEM2 | -1.143 |  |  |
| RCL1 | -1.137 |  |  |
| S100A16 | -1.123 |  |  |
| COMMD2 | -1.120 |  |  |
| IMPA1 | -1.112 |  |  |
| PLIN5 | -1.103 |  |  |
| CPEB4 | -1.096 |  |  |
| NES | -1.091 |  |  |
| NOS3 | -1.090 |  |  |
| FAM175A | -1.087 |  |  |
| C1orf64 | -1.086 |  |  |
| C12orf23 | -1.080 |  |  |
| F2RL1 | -1.061 |  |  |
| CPXM1 | -1.056 |  |  |
| PALLD | -1.056 |  |  |
| ARG2 | -1.055 |  |  |
| ZFP36 | -1.051 |  |  |
| ATXN1 | -1.045 |  |  |
| TDG | -1.034 |  |  |
| MAP1LC3B | -1.028 |  |  |
| CD3D | -1.025 |  |  |
| C8orf4 | -1.018 |  |  |
| KRT19 | -1.008 |  |  |

**Supplementary Table S3. List of primers for qRT-PCR and sequencing reactions**

| **Primers for qRT-PCR** |  |
| --- | --- |
| **Name** | **Primer** |
| SF3B3_sense | CTGCAGTACACACCCAGGAA |
| SF3B3_antisense | CCATCTGCTGCTTTCTCTGA |
| GOLGB1_sense | TTGTGAGGACAAGTGAAGCAA |
| GOLGB1_antisense | TGAAAGAACAGGGACTCTTAAACA |
| 18s_sense | GGATGTAAAGGATGGAAAATACA |
| 18s_antisense | TCCAGGTCTTCACGGAGCTTGTT |
|  |  |
| **Primers for construction of expression vectors** | |
| **Name** | **Primer** |
| SF3B3-PCDNA3 sense | CAGTGTGCTGGAATTCATGTTTCTGTACAACTTAACCTTGCAGAG |
| SF3B3-PCDNA3 antisense | AATAGGGCCCTCTAGAGAAGGCGTAGCGGGTCC |
| Head brick of GOLGB1-PCDNA3 sense | CAGTGTGCTGGAATTCATGCTGAGCCGATTATCAGGAT |
| Head brick of GOLGB1-PCDNA3 antisense | CCATTTCTTGCTCAGCTTCC |
| Tail brick of GOLGB1-PCDNA3 sense | GGAAGCTGAGCAAGAAATGG |
| Tail brick of GOLGB1-PCDNA3 antisense | AATAGGGCCCTCTAGACTATAGATGGCCCGTAAAACACAGAATG |
|  |  |
| **Primers for site-directed mutagenesis** |  |
| **Name** | **Primer** |
| SF3B3 mutation sense | TTGAAACGGACTACAATGCCTAC |
| SF3B3 mutation antisense | GTAGGCATTGTAGTCCGTTTCAA |
| GOLGB1 mutation sense | TGGAGAATAGCTTGTCTCACCTTG |
| GOLGB1 mutation antisense | TGAGACAAGCTATTCTCCACAGT |
|  |  |
| **Primers for sequencing reaction** |  |
| **Name** | **Primer** |
| CMV_seq | CGGTGGGAGGTCTATATAAGCAG |
| BGH_seq | TAGAAGGCACAGTCGAGG |
| GOLGB1_Seq_1 | GCAGAACAACCTGCACAGAG |
| GOLGB1_Seq_2 | AGTCTGCCTGTGATGCTCTAAA |
| GOLGB1_Seq_3 | TTTCCCTTAATGCCAAATGAA |
| GOLGB1_Seq_4 | AGGGGGCAGAACGTGTAAG |
| GOLGB1_Seq_5 | CAGCATATAAGGAAGGATTTGGA |
| GOLGB1_Seq_6 | GGAACTTCTGTTGCCCAGAT |
| GOLGB1_Seq_7 | TGGAAAGCCAAGTTTCTGCT |
| GOLGB1_Seq_8 | GACTCTCTAAGTGAAGAGGTTCAAGA |
| GOLGB1_Seq_9 | AGCAGCAGTTAGTCAAGGAAAAA |
| GOLGB1_Seq_10 | TGTCTTCCCTCCAGGATGAT |
| GOLGB1_Seq_11 | CCCAGGAGGAAGAGGAGAAT |
| GOLGB1_Seq_12 | TGCTTTGTTTTCCTCCTCTCA |
| GOLGB1_Seq_13 | ACCTGTGGAATGAGCTGGAG |
| SF3B3_F_Seq_1 | TCCAATGTTTGCTTGTCTGG |
| SF3B3_F_Seq_2 | AAACCTTGTGCTGGTTGATG |
| SF3B3_F_Seq_3 | GTCTCGCTTCCTGGCTGT |
| SF3B3_F_Seq_4 | CAGATGGCAGAGGAAATGGT |
| SF3B3_R_Seq_5 | GATCGGGGGTTTAGTATCAGG |
